# Structural and Biophysical Investigation of the Key Hotspots on the Surface of Epstein–Barr Nuclear Antigen 1 Essential for DNA Recognition and Pathogenesis

**DOI:** 10.3389/fmolb.2021.664436

**Published:** 2021-06-29

**Authors:** Huma Farooque Hashmi, Muhammad Waseem, Syed Shujait Ali, Zahid Hussain, Kaoshan Chen

**Affiliations:** ^1^ College of Life Sciences, Shandong University, Jinan, China; ^2^ Faculty of Rehabilitation and Allied Health Science, Riphah International University, Islamabad, Pakistan; ^3^ Center for Biotechnology and Microbiology, University of Swat, Swat, Pakistan

**Keywords:** EBNA1, DNA recognition, MD simulation, in silico mutagenesis, free energy calculations

## Abstract

Epstein-Barr Virus (EBV) is considered the most important human pathogen due to its role in infections and cellular malignancies. It has been reported that this Oncolytic virus infects 90% world’s population. EBNA1 is required for DNA binding and survival of the virus and is considered an essential drug target. The biochemical and structural properties of this protein are known, but it is still unclear which residues impart a critical role in the recognition of dsDNA. Intending to disclose only the essential residues in recognition of dsDNA, this study used a computational pipeline to generate an alanine mutant of each interacting residue and determine the impact on the binding. Our analysis revealed that R469A, K514A, Y518A, R521A and R522A are the key hotspots for the recognition of dsDNA by the EBNA1. The dynamics properties, i.e. stability, flexibility, structural compactness, hydrogen bonding frequency, binding affinity, are altered by disrupting the protein-DNA contacts, thereby decreases the binding affinity. In particular, the two arginine substitution, R521A and R522A, significantly affected the total binding energy. Thus, we hypothesize that these residues impart a critical role in the dsDNA recognition and pathogenesis. This study would help to design structure-based drugs against the EBV infections.

## Introduction

Due to the prevalent infections instigated by Herpes viruses, it is considered as an important virus in human pathogens flora. This diverse pathogenic flora Epstein-Barr Virus (EBV) is regarded as the most important human pathogen due to its role in infections and cellular malignancies (Bochkarev et al., 1996; Yasuda et al., 2011) It has been reported that this Oncolytic virus infects 90% world’s population. Immortalization of B lymphocytes accompanies the main EBV infection and stimulates them to replicate as lymphoblastic cell lines (Garai-Ibabe et al., 2012). Alongside the B lymphocytes infection, EBV also causes infectious mononucleosis by targeting the epithelial cells (Pope et al., 1968; Haque and Crawford, 1996). Nasopharyngeal carcinoma (NFC), muscle cell sarcoma and gastric carcinomas (GaCa), Hodgkin’s lymphoma (HL), Burkitt’s lymphoma (BL), extranodal lymphoma of T/NK cell origin and post-transplant lymphoproliferative disease (PT-LPD) are among the EBV associated diseases (Taylor et al., 2015). EBV caused tumors stores the viral genome as a multi-copy episome in the nucleus of infected cells (Tikhmyanova et al., 2014). During the latent infection, progenitor virions are not reproduced, but alternatively, a set of genes essential for survival and proliferation are expressed (Matsuura et al., 2010). Epstein-Barr Nuclear Antigen 1 (EBNA1) acts to preserve the latent viral genome in proliferating cells (Okano, 1998). This protein is expressed in the malignant cell and sustain the proliferation (Leight and Sugden, 2000).

The EBV genome encodes ∼100 genes, among which EBNA1 is the key nuclear antigen that works with the other five others (Hiraku et al., 2014). EBNA1 is almost detected in every kind of infection induced by the EBV in both latent and lytic infections (Gianti et al., 2016). This essential antigen is reported to be involved in mitotic segregation of episomes, replication, reactivation, viral transcription, and lytic infection of EBV (Young and Rickinson, 2004). It has also been reported that EBNA1 possess a similar structure to that of human papillomavirus (HPV) E2 protein and the Kaposi’s Sarcoma Associated Herpesvirus (KSHV) LANA protein (Garber et al., 2002). In addition to structural similarity, these proteins are reported to have similar function, i.e. DNA binding and episome regulation (Wang et al., 2006). The biochemical and structural properties of this protein are known. EBNA1 works as a dimer with two functional domains (Pope et al., 1968; Haque and Crawford, 1996). The two terminals CTD (carboxy-terminal DNA-binding domain) and ATCTD (amino-terminal chromosome tethering domain) bind the 18ps DNA to initiate the plasmid and viral genome replication (Lindner and Sugden, 2007). Due to the multi-faceted role of EBNA1, it is the primary drug target for the treatment of EBV associated infections.

The crystallographic structure of ENBA1 has been solved, and reported that 459–607 residues at C-terminal are required for DNA binding (Bochkarev et al., 1998). Previous studies determining the dissociation constant (KD) for the EBNA1-DNA association reported that mutating interacting residues, R469A reduced the binding of DNA by 300-fold, Y518A by 80-fold and R522A by 1600-fold (Cruickshank et al., 2000). Additionally, other studies also reported that K514A also reduces the binding affinity significantly. At the same time, others reported that three residues R491E, R491A, and D581E significantly impair the DNA binding (Morgunova et al., 2015). These studies mutated only selected residues, while the impact of others remains a question. To understand each residue’s impact and reveal only a few residues that are required explicitly for DNA recognition while others are supplementary interactions, an in-depth investigation is needed. To disclose only the essential residues, this study used a computational pipeline to generate an alanine mutant of each interacting residue and determine the impact on the binding. Highly destabilizing and affinity reducing mutations were subjected to biophysical investigation to reveal their real effect on the binding. Our analysis would help to target the critical hotspots for future rational and structure-based drug designing to curtail the EBV associated lytic and latent infections.

## Materials and Methods

### Structure Retrieval and Preparation

The RCSB protein databank (http://www.rcsb.org/) repository was accessed for structural retrieval. Structural deformities were detected and addressed (Rose et al., 2016). The missing hydrogens were added, and partial charges were assigned. The structure was also analyzed for structural breaks and unknown residues. The structure was minimized and prepared before *in silico* mutagenesis and molecular docking.

### Epstein-Barr Nuclear Antigen 1-DNA Docking

For the docking HADDOCK (High Ambiguity Driven protein-protein Docking) (Dominguez et al., 2003) was utilized. It uses biochemical and structural data to drive the docking process. The Guru interface with approximately 500 features considered as the best to predict the docking poses. Using default parameter i.e. lowest intermolecular energies, the best structural complex was extracted. We also used NPDock (Tuszynska et al., 2015), an online web server, which uses the scoring of poses, clustering of the best-scored models, and refinement of the most promising solutions to give the best results. The best scoring complex was retrieved from NPDock and analyzed. To determine the interaction of different residues with the DNA DNAproDB (Sagendorf et al., 2020) was used to extract the interactions from DNA-nucleic acid complexes.

### Interface Analysis and Mutants Library Construction

Using the machine learning protocol implemented in MOE (Vilar et al., 2008) the Alanine scanning approach was applied to compute the impact of each residue in the interaction with the DNA. The dAffinity and dStability parameters are essential considerations in the ASM (alanine scanning module) which determine the relative stability and affinity changes upon substitution. The detailed mechanism of this alanine scanning mutagenesis and residue scan approaches has been discussed previously (Junaid et al., 2019). Furthermore, we also used mCSM-NA an online webserver, for the affinity changes prediction upon the alanine substitution uses the graph-based signature model. Residues with high-affinity changes were subjected to molecular dynamics simulation investigation.

### Molecular Dynamics Simulation

To further provide deep insight into the stability and affinity changes upon the alanine substitution, the dynamic features of each complex was determined using the AMBER 20 simulation package. For protein ff14SB, while for DNA, the OLS3 force field was utilized (Salomon-Ferrer et al., 2013). With the TIP3P water model containing 9,784 water molecules, each complex was solvated at 10.0 Å. A total of 29 sodium ions were added to neutralize each system. Multistep energy minimization each 6,000 steps and 3,000 steps of conjugate gradient minimization were completed. Keeping the heating parameters default 300 K for 200 ps, each complex was heated. For density equilibration, using weak restraint for 2 ns at constant pressure was executed. Finally, 200 ns MD using constant pressure was achieved. Langevin thermostat with 1 atm pressure and 300 K for temperature control (Zwanzig, 1973), while Particle Mesh Ewald (PME) algorithm to evaluate long-range interactions respectively (Ryckaert et al., 1977; Roe and Cheatham, 2013) with the cutoff, distances 10 Å. For the covalent bonds involving hydrogen, the SHAKE algorithm was used (Ryckaert et al., 1977). All the simulations were GPU accelerated.

### Post-Simulation Analyses

The thermodynamics state function, i.e. RMSD, residual flexibility, i.e. RMSF, structural compactness, i.e. radius of gyration (Rg) and the total number of hydrogen bonds over the simulation were computed by using CPPTRAJ and PTRAJ modules integrated with AMBER (Roe and Cheatham, 2013).

### Binding Affinity Calculations

To connect the alanine mutations with the binding affinity changes, the binding free energy of each alanine substituted complex was determined. The free energy scoring function (MMGBSA) is an extensively used approach to evaluate the free energy of a protein-ligand, protein-protein and protein-nucleic acids (Khan et al., 2018; Ali et al., 2019; Khan et al., 2019; Khan et al., 2020a; Khan et al., 2020b; Khan et al., 2020c; Khan et al., 2020d; Khan et al., 2020e; Khan et al., 2020f; Hussain et al., 2020). It used the following equation to calculate the free energy.

$$\Delta G_{bind}=\Delta G_{complex}-\left[\Delta G_{receptor}+\Delta G_{ligand}\right]$$

Each term in the such as electrostatic, van der Waals interactions, polar and nonpolar were predicted using the following equation:

$$G=G_{bond}+G_{ele}+G_{vdW}+G_{pol}+G_{npol}$$

Clustering of MD trajectories using PCA and Free Energy Landscape (FEL).

An unsupervised learning approach known as Principal Component Analysis to describe the motion of MD trajectories (PCA) (Pearson, 1901; Wold et al., 1987) and gain information about the internal motion of the system using CPPTRAJ. For the eigenvector and their atomic coordinates, the spatial covariance matrix was calculated. A diagonal matrix of eigenvalues was generated using the orthogonal co-ordinate transformation. The Principal Components were derived based on the eigenvectors and eigenvalues. The predominant movements during the simulation were plotted using these PCs. (Balsera et al., 1996; Ernst et al., 2015). Furthermore, Free energy landscape (FEL) was constructed to capture the different energy minima at different simulation time.

## Results And Discussion

### Structure Retrieval and Epstein-Barr Nuclear Antigen 1-DNA Docking

**FIGURE 1:**
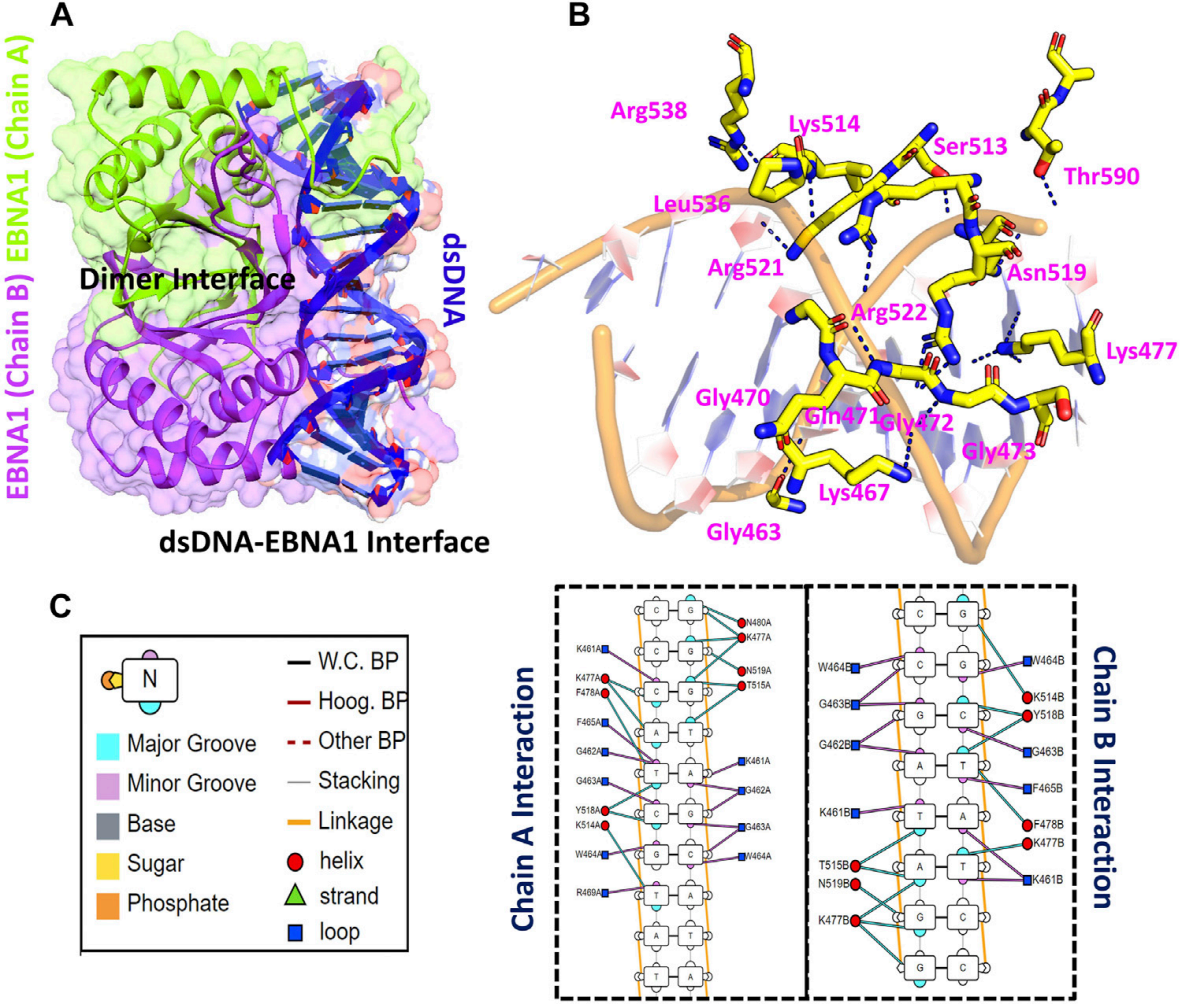
Represent the crystallographic structure of EBNA1-DNA. **(A)** shows the dimer binding to the dsDNA. Both chains are coloured differently. **(B)** shows the 3D interactions of EBNA1 and DNA. **(C)** shows the 2D interactions of and the legend of the interaction pattern. The legend shows the respective interaction between the DNA and EBNA1. The circle in red colour represent the interaction with helix; navy blue colour represent the loop, while the cyan triangle shows the strand. Additionally, the minor groove, major groove and nucleotides are also coloured differently.

Using accession number 5T7X the structure of the EBNA1 was retrieved. The structure is a dimer interface of two EBNA1 chains and 18bps DNA. The structural representation of the EBNA1-DNA complex is shown in Figure 1A. HADDOCK predicted the correct docking conformation with the binding energy -295.63 kcal/mol. The interactions predicted by the DNAproDB showed that G462, G463, W464, F465, R469, N475, K477, F478, R491, K514, Y518, R521 and R522 are involved in interaction with the DNA. The 3D interaction of these residues with the DNA is given in Figure 1B. A different number of hydrogen bonds were formed by each residues ranging from one to five at the interface. The specific hydrogen bonding interactions are shown in Figure 1C. These residues contributed to the total binding energy. To potentially determine the impact of each of this residue, alanine scanning revealed its impact on the binding of DNA. Among the 13 residues at the interface G462A and G463A increase the binding affinity while the remaining 11 residues decrease the binding affinity at different folds.

As tabulated in Table 1, it can be seen that R469A, K514A, Y518A, R521A and R522A significantly affected the binding of DNA as compared to others. In the case of the R469A, the predicted ΔG was reported to −5.784 kcal/mol, while the dAffinity was also predicted to be reduced (−1,009.21 kcal/mol). The predicted ΔG for K514A was reported to be −3.638 kcal/mol, while the dAffinity was reported to be −1,021.32 kcal/mol, respectively. For the Y518A the predicted ΔG was -3.406 kcal/mol; however, the dAffinity was reported to be −1,020.32 kcal/mol. Intriguingly the dAffinity for R521A and R522A was comparable. The predicted dAffinity for R521A and R522A was reported to be −1,009.60 kcal/mol and −1,009.37 kcal/mol, respectively. Furthermore, the predicted ΔG for R521A was −5.866 kcal/mol, while for R522A, it was −6.008 kcal/mol. In the EBNA-DNA co-crystal structure, the three targeted amino acids are oriented toward the DNA but are too far from the nearest H-bond acceptor in the bases (more than 6 Å) to form H-bonds. Hence, these results also show that R469A, K514A, Y518A, R521A and R522A are required for DNA recognition and are the key hotspots for drug discovery. Thus, these residues were selected for further evaluation and subjected to molecular dynamics simulation to understand its dynamics behaviour and reveal its binding energy differences.

**TABLE 1:** The table shows the alanine scanning results of the interacting residues. dStability, dAffinity, predicted ΔG and the outcome of each mutation upon substitution is given. Highly affinity reducing mutations are given as bold and were subjected to molecular dynamics simulation-based investigation. All the energies are given in kcal/mol.

Index	Mutant residue	dStability^a^	dAffinity^b^	Predicted ΔG^c^	Outcome
1	G462A	−161.94	−1,297.62	1.482	Increased affinity
2	G463A	−152.55	−1,021.36	1.488	Increased affinity
3	W464A	−153.84	−1,022.16	−1.656	Reduced affinity
4	F465A	−158.65	−1,022.34	−2.878	Reduced affinity
**5**	**R469A**	−**154.65**	−**1,009.21**	−**5.784**	**Reduced affinity**
6	N475A	−163.71	−1,016.41	−0.592	Reduced affinity
7	K477A	−160.67	−1,020.26	−0.824	Reduced affinity
8	F478A	−161.56	−1,022.69	−2.148	Reduced affinity
9	R491A	−225.26	−1,012.93	−1.986	Reduced affinity
**10**	**K514A**	−**165.16**	−**1,021.32**	−**3.638**	**Reduced affinity**
**11**	**Y518A**	−**156.59**	−**1,020.47**	−**3.406**	**Reduced affinity**
**12**	**R521A**	−**158.75**	−**1,009.60**	−**5.866**	**Reduced affinity**
**13**	**R522A**	−**156.41**	−**1,009.37**	−**6.008**	**Reduced affinity**

a **dStability =** it is the relative stability change upon the mutation. The more the negative the more instable the structure.

b **dAffinity =** it is the relative affinity change between the wild type and mutated complex. Negative dAffinity means the mutation will increase the binding affinity while positive dAffinity mean it will decrease the binding affinity.

c **Predicted ΔG** = it implies a similar formula but different algorithm to calculate the binding differences between the wild type and mutant. ΔG shows change in the binding free energy changes upon the mutation.

Bold values are Mutations selected for MD simulation.

### Mutation Stability Correlation (Root Mean Square Fluctuation)

**FIGURE 2:**
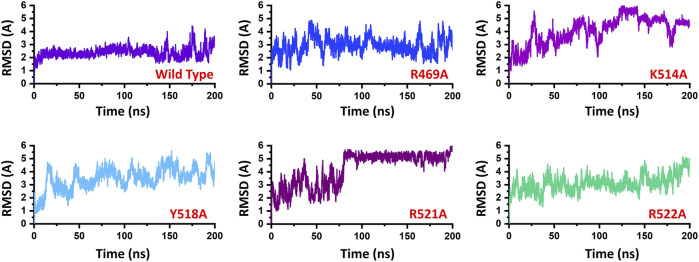
Represent the dynamic stability of EBNA1-DNA bound wild type and mutant complexes. All the complexes are coloured differently and tagged. The *x*-axis shows the time in nanoseconds, while the *y*-axis shows RMSD in Å.

**FIGURE 3:**
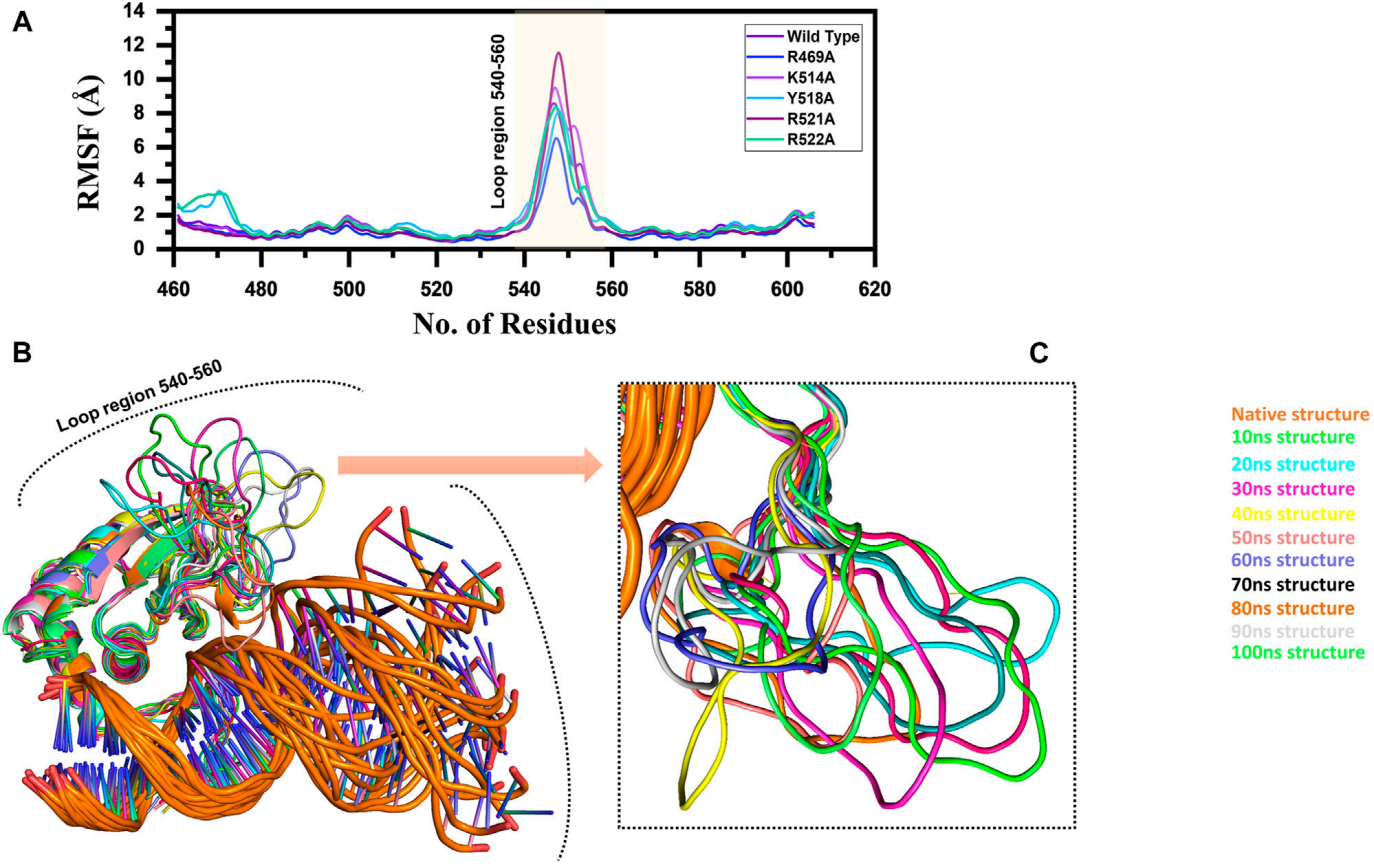
**(A)** Represent the residual flexibility of EBNA1-DNA bound wild type and mutant complexes. All the complexes are coloured differently and tagged. The *x*-axis shows the total number of residues, while the *y*-axis shows RMSF in Å. **(B)** and **(C)** shows the highly fluctuated regions on the protein’s structure.

To demonstrate the mutation’s stability correlation, the thermodynamics state function Root mean square fluctuation (RMSD) of the wild type and the mutant complexes was calculated as a function of time. A 200 ns simulation trajectory for each complex was analyzed. Results for all the complexes are presented in Figure 2. In the case of the wild type, the structure gained stability at 2.0 Å. The structure remained remarkably stable during the simulation. After reaching 150 ns the structure converged, and the RMSD increased which is due to the loop opening and closing surrounded the DNA. It was observed that a loop region between 540–560 deviated from its mean structure significantly and thus the RMSD fluctuated substantially. Furthermore, the terminal of the DNA molecule packs the protein by opening and closing also causes significant structural deviation from its mean position thus causes structural destability. This can be inferred from Figure 3A where the loop region in all the complexes fluctuated significantly and Figures 3B,C shows the closer look into the loop region which is significantly deviated at different intervals. In the case of the R469A mutation, the complex experiences significant divergence from the initial structure. The equilibrium was never achieved during the 200ns simulation time. During the first 50ns simulation the structure owned significant convergence to the following 100 ns. Between 50 and 150 ns the RMSD remained lower and experienced only one significant convergence at 100 ns. Afterwards, the structure remained unstable until the 200 ns. The average RMSD for R469A was reported to be 3.5 Å. The K514A mutant, which is considered as an important residue for the DNA binding, caused significant perturbation upon the substitution. The complex remained significantly unstable during the 200 ns simulation time. The average RMSD for the first 25 ns remained 3.0 Å. Until the first 25 ns the RMSD remained 2.0 Å; however, a significant convergence was observed abruptly, and this trend continues until 200 ns. On the other hand, the Y518A behaviour was also comparable with the K514A. Significant convergence at different intervals was reported over the simulation and the average RMSD remained 4.0 Å. Furthermore, the two arginine replacements at position R521 and R522 significantly altered the dynamics and interaction of the EBNA1-DNA. These replacements caused significant destabilization of the EBNA1-DNA complex at different interval of the 200 ns. These residues are also reported experimentally to cause significant instability of the complex. The average RMSD remained higher for R521A (5.5 Å), while the average RMSD for R522A remained lower but converged significantly. The RMSD continues to increase during the last 50 ns. Thus these results suggest that the wild type structural topology is required for stable interactions, and the mutation-induced here does not only affect the binding of the complex but also the stability. Hence further study on the impact of the substitution justified the effect of these residues on the binding of dsDNA and its druggability properties.

### Residual Flexibility of the Wild Type and Mutant Complexes

Furthermore, to connect the residual flexibility with these substitutions, we estimated RMSF (root mean square fluctuation). The wild and mutant complexes owned comparable flexibility levels. It can be seen that all the structures possess a more similar pattern of flexibility. The residues 460–480, particularly in Y518A and R522A possess more flexible behaviour than the others, which is explained in Figure 8 that it deviated more than the mean point and the mutations cause an allosteric effect on the flexibility. Significant residual flexibility for region 530–560 can be observed. These results show that complexes possess more rigid structures. The RMSF results for all the complexes are represented in Figure 3A. However the loop region which causes structural perturbation and flexibility is shown in Figures 3B,C.

### Hydrogen Bonding Analysis of the Wild Type and Mutant Complexes

Furthermore, to understand the impact of these substitutions on the total number of hydrogen bonds, we calculated the total number of hydrogen bonds during the 200 ns trajectories and the bonding network between the EBNA1 and DNA. Hydrogen bonding rearrangement was observed during the simulation. Among the key bonding in the wild type R469 residue formed extra two interactions with T11 and A28. Among the others, R522 formed one additional hydrogen bonds with 1.83 Å. Initially, a total of 15 bonds were observed, while after simulation with these three extra interactions formed and the total bonds were reported to be 18 in total in wild type. On the other hand, the R469A lost multiple interactions during the simulation, particularly those formed with R469 residue, consequently remaining 13 hydrogen bonds between EBNA1 and DNA. Among these, Lys461, Arg521 and Arg522 created multiple interactions while the other residues were involved in single interaction only. This shows that in the wild type complex, R469 formed three interactions while those are lost here signifies its role in recognition.

**FIGURE 4:**
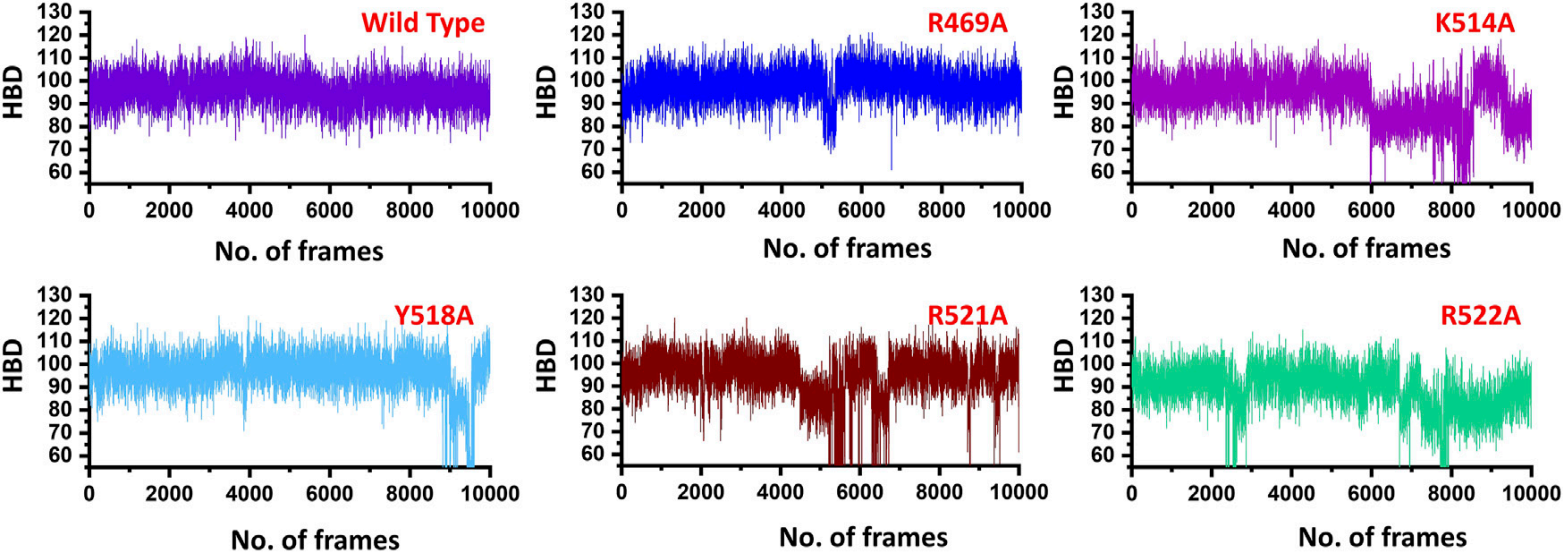
Show the total number of hydrogen bonds in the EBNA1-DNA bound wild type and mutant complexes. All the complexes are coloured differently and tagged. The *x*-axis shows the total number of frames, while the *y*-axis shows the total number of hydrogen bonds. Structural compactness of the wild type and mutant complexes.

Similarly, only 11 hydrogen bonds were observed in K514A complex. Among the hydrogen bonding interaction, K514 lost its interaction while R521 and R522 also lost three interactions during the simulation. This shows that the mutation has allosterically affected the other residues and destabilized the interaction with the DNA molecule. Moreover, with 12 hydrogen bonds between EBNA1 (Y518A) and DNA complex R469, R521 and R522 lost their multiple interactions which were reported to be sustained in wild type complex. Furthermore, the two essential residues R521A and R522A which significantly contributed to the total binding energy reported in substantial hydrogen bonds reduction between EBNA1 and DNA. In the case of R521A only 10 hydrogen bonds were reported, while only nine bonds were reported between R522A and DNA. In R522A five bonds formed by R521 and R522 are lost. This shows that the two arginine moieties play a significant role in recognition of DNA. After all, the average number of hydrogen bonds were calculated for each complex. As given in Figure 4, a significant drop can be observed in the mutant complexes, particularly in the K514A, R521A and R522A complexes. The average number of hydrogen bonds in the wild type was reported to be 98, while in the mutant complexes (R469A), the H-bonds were reported to be 94, (K514A) H-bonds were observed to be 90, Y518A reported 94, while the significant drop was observed in R521A (86) and R522A the total H-bonds were 88. Thus it can be seen that upon substitution, H-bonds count was decreased and thus, these residues potentially act as druggable hotspots. The hydrogen bonding results for all the complexes are represented in Figure 4.

**FIGURE 5:**
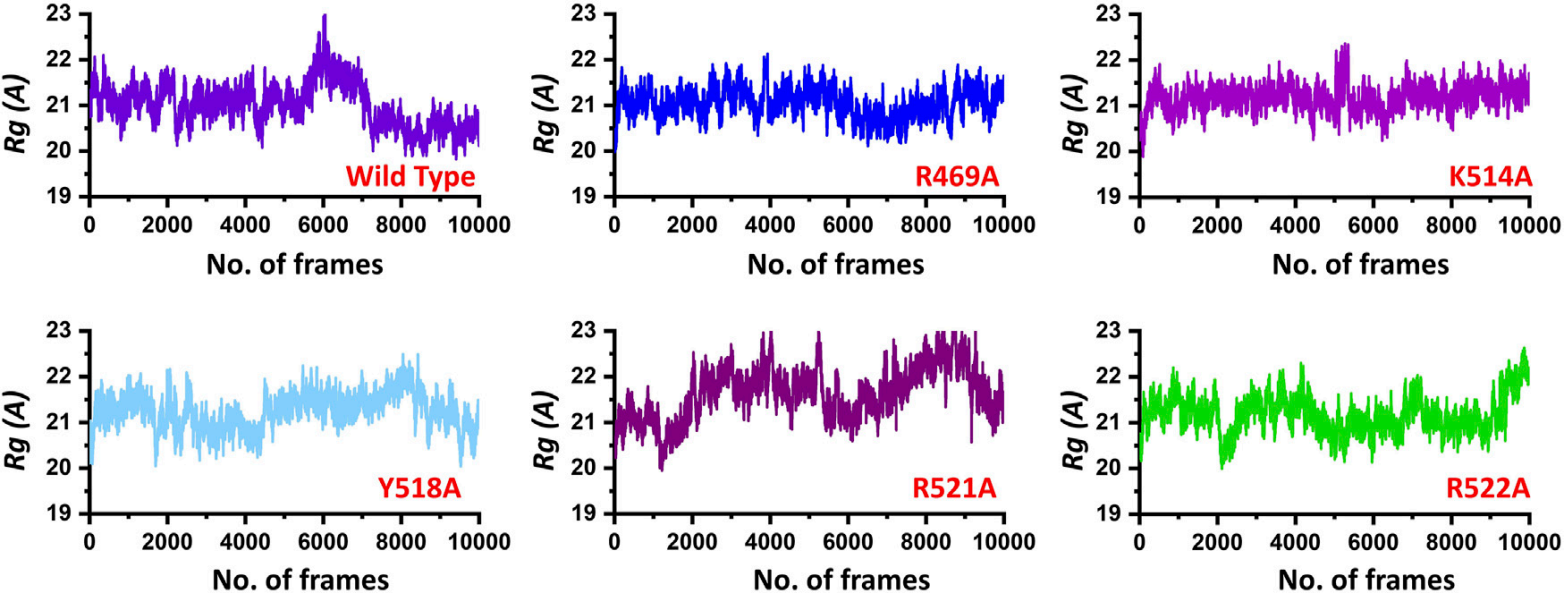
Show the Rg (radius of gyration) of the EBNA1-DNA bound wild type and mutant complexes. All the complexes are coloured differently and tagged. The *x*-axis shows the total number of frames, while the *y*-axis shows *Rg* (radius of gyration).

Next, to connect the protein conformation changes with the compactness of each complex, we calculated Rg (radius of gyration) as a function of total frames in a trajectory. In the case of the wild, the complex remained more compact than the others. The average Rg for the wild type was reported to be 21.0 Å. In the case of R469A, the same pattern was observed. The results of R469A and wild type was comparable. On the other hand, the K514A the Rg remained higher during the simulation time period. During the first 100 ns. the Rg was observed to be higher, which significantly increased between 100 and 125 ns. Afterwards, the Rg remained uniform. In the case of Y518A, the structural compactness also remained haphazard. Initially, it remained higher but then decreased between 40 and 100 ns while then increased and decreased continuously until the 200 ns. In the case of R521A and R522A, the structural compactness is disrupted significantly. This shows that the loss of structural compactness is due to the binding and unbinding events that occurred during the simulation, and this can be clearly concluded from Figure 4 as the total number of hydrogen bonds are vary in numbers. Besides the packing of EBNA1 by the DNA terminal and the opening and closing of the loop region 530–560 also demonstrates the compactness variations. The calculated Rg (radius of gyration) results for all the complexes are represented in Figure 5.

### Principal Motions of the Wild Type and Decoy Designed Peptides

**FIGURE 6:**
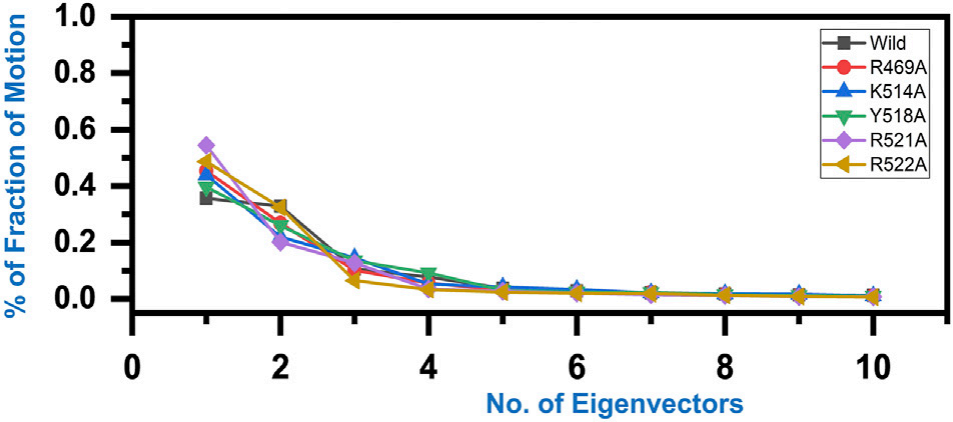
Fraction of the first ten eigenvectors indices generated from the MD trajectory. The percent contribution of each eigenvector is plotted against the corresponding eigenvector.

Variations in the proteins’ trajectories motions were exhibited by each system was captured through PCA. PCA would help to comprehend conformational changes induced variations in the proteins’ motion of the wild type and mutant complexes. The internal motion was shown by the first three eigenvectors, while localized fluctuations in the remaining eigenvectors in each complex were observed (Figure 6). In the case of wild type peptide complex, the first three eigenvectors contributed 35% variance to the total observed motion, while in R49A, 45%, K514 43%, Y518A 39%, R521A 54% while R522A accounted for 48% variance in motion. This shows the increased motion in the mutated systems and may explain the structural rearrangement due to the mutations in the binding site.

**FIGURE 7:**
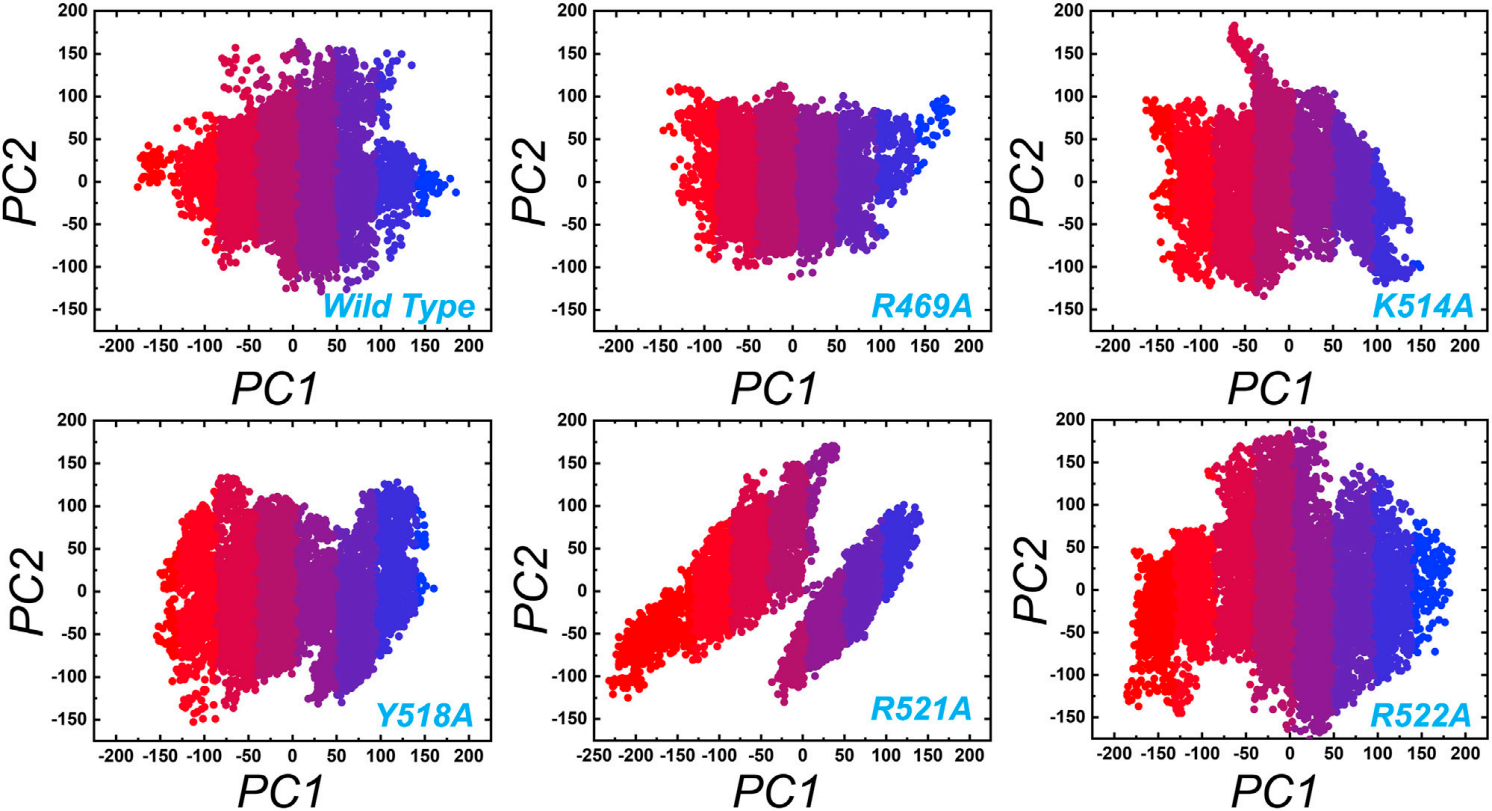
Principal component analysis (PCA) of wild type and the mutant complexes of the EBNA1-DNA. Two PCs i.e. PC1 and PC2 were used for the scattered plot. Each panel represent the respective complex as tagged.

In order to obtain plausibly attributed movements, the first two eigenvectors were projected against one another. The continuous representation of the red to blue colour indicates the transition from one conformation to another over the simulation period. The dots, starting with red and ending in blue, represent each frame. In each complex periodic jumps and continuous overlapping can be observed (Figure 7). consequently, all these annotations infer that mutations expressively affected the structure and variations in the internal dynamics of the complexes.

**FIGURE 8:**
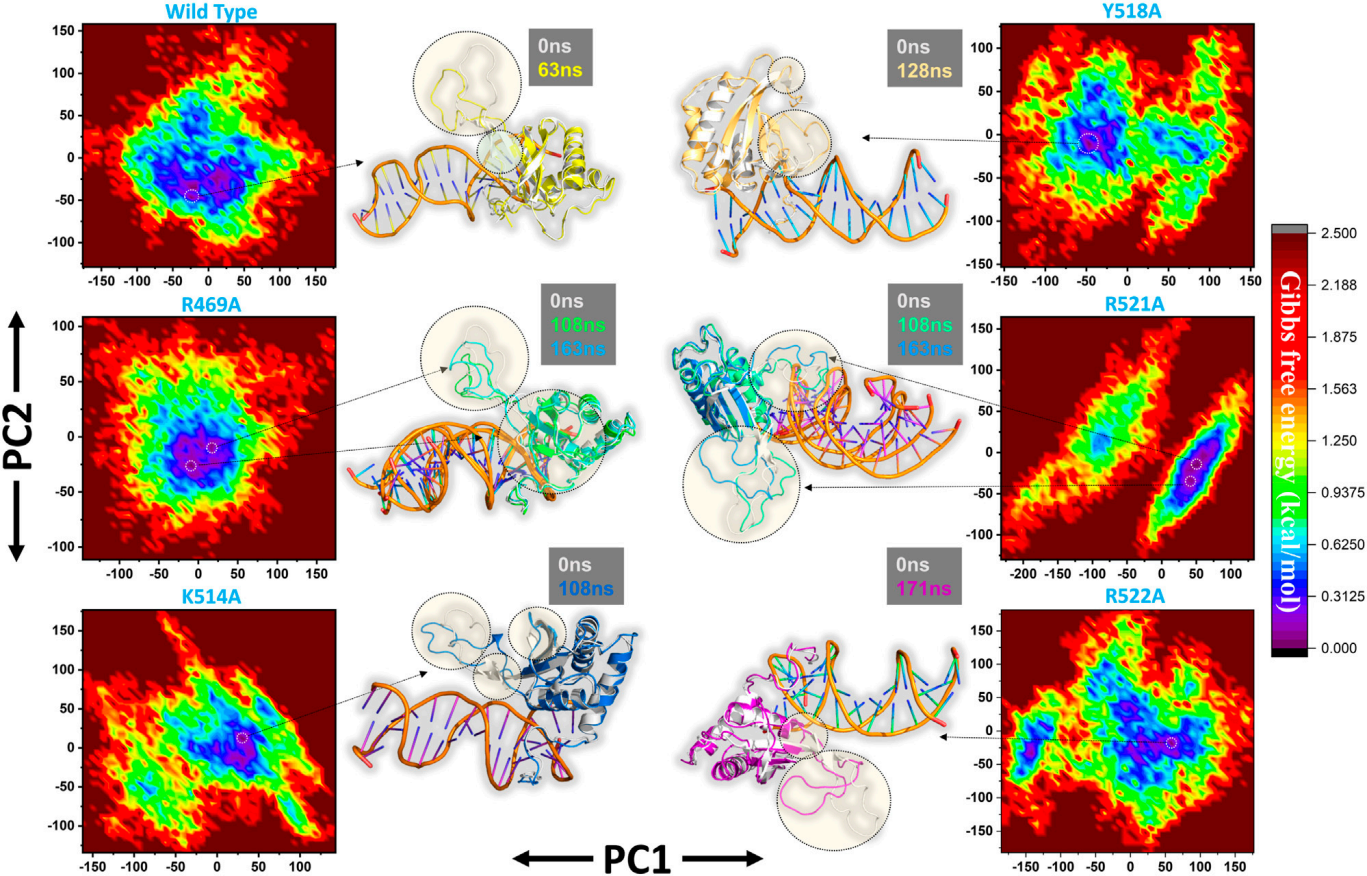
Free Energy Landscape (FEL) of all the complexes i.e. wild type, R469A, K514A, Y518A, R521A and R522A. The first PC1 and second PC2 from the PCA of the backbone carbon were used.

Furthermore, a free energy landscape (FEL) was constructed to relate the structural features and thermodynamics properties. To obtain the energy minima based on probability of given data points of MD trajectories and to map the minimum energy conformation of the all the complexes during the explored time scale, and finally to connect the structural changeovers between these minima. Figure 8 represent the FEL of all the complexes i.e. wild type, R469A, K514A, Y518A, R521A and R522A. The wild type, K514A, Y518A and R522A shows only one energy minima while R469A and R521A exhibit two lowest energy minima separated by a small subspace, thus explaining global conformational variations adjusted by the mutant complexes in response to mutations. The major variations in these conformations were the loop deviation and nearby beta-sheets conversion. All the variations are highlighted in the Figure 8.

### Binding Free Energy Calculations

To further connect the protein conformation changes with the binding affinity, we calculated the total binding energy using MM-GBSA approach. This method is considered as the best tool for calculating the real time-binding energy of the biological macromolecules complex. Herein to estimate the impact of the alanine substitutions at a specific position, we estimated the binding free energy. As given in Table 2, significant differences in the binding energy can be observed. The electrostatic contribution is significant in each complex. In the wild complex, the total binding free energy was estimated to be −145.18 ± 0.269 kcal/mol. However, in the R469A, this total binding energy was calculated to be −110.75 ± 0.230 kcal/mol. This reduction in the binding energy is due to the disruption of the hydrogen bonding network caused by the alanine substitution. A particular interaction formed by the minor groove of DNA with the loop residue R469 causes a significant decline in the total binding energy. The total binding energy results of K514A and Y518A mutant complexes are comparable. For the K514A complex, the total binding energy was reported to be −104.97 ± 0.175 kcal/mol while the total binding energy for Y518A was −102.17 ± 0.190 kcal/mol. This is due to the loss of three hydrogen bonds formed by helix residues with the major groove are diminished upon the substitution. In case of K514A only one hydrogen bond while in case of Y518A, two important hydrogen bonds are lost. Significant drop out was observed in the total binding energy of the R521A mutant complex. The TBE was for R517A was reported to be −97.93 ± 0.226 kcal/mol. On the other hand, the estimated binding energy for R522A was −100.04 ± 0.215 kcal/mol. Overall, these results show that the mutations induced significant energy drop out but the R521A and R522A reduced the binding energy by many folds. Hence these residues play a vital role in recognition of dsDNA and contribute to the infection initiation and progression.

**TABLE 2:** Display the total binding energy of the wild type and mutant complexes. Van Der Waal forces, electrostatic energy, generalized born, surface area and the total binding energy values for each complex is given. All the energies are calculated in kcal/mol.

Complex name	vdW	Electrostatic	EGB	ESURF	Total ΔG
Wild type	−132.12 ± 0.142	−4,900.21 ± 2.54	4,904.28 ± 2.43	−17.12 ± 0.017	−**145.18** ± 0.269
R469A	−124.36 ± 0.165	−4,210.70 ± 2.300	4,240.52 ± 2.204	−16.21 ± 0.015	−**110.75** ± 0.230
K514A	−131.48 ± 0.127	−4,331.61 ± 2.0239	4,375.12 ± 1.967	−17.00 ± 0.010	−**104.97** ± 0.175
Y518A	−127.19 ± 0.137	−5,030.86 ± 2.253	5,072.45 ± 2.198	−16.56 ± 0.013	−**102.17** ± 0.190
R521A	−132.93 ± 0.1584	−4,343.05 ± 2.353	4,395.21 ± 2.299	−17.16 ± 0.016	−**97.93** ± 0.226
R522A	−132.66 ± 0.187	−4,420.46 ± 2.280	4,469.95 ± 2.241	−16.87 ± 0.019	−**100.04** ± 0.215

Bold values are the total binding energy.

## Conclusion

In conclusion, herein, we systematically investigated the mechanism of dsDNA recognition by the EBNA1 protein. Our analysis revealed that R469A, K514A, Y518A, R521A and R522A are the key hotspots for drug discovery against the various tumors caused by EBV. In particular, the two arginine substitution R521A and R522A, significantly affected the total binding energy. Thus we hypothesize that these residues impart a critical role in the dsDNA recognition and pathogenesis. This study would help to design structure-based drugs against EBV infections.

## Data Availability

Publicly available datasets were analyzed in this study. This data can be found here: http://www.rcsb.org/, the accession number(s) can be found in the article/Supplementary Material.
